# *In-Silico *screening of Pleconaril and its novel substituted derivatives with Neuraminidase of H1N1 Influenza strain

**DOI:** 10.1186/1756-0500-5-105

**Published:** 2012-02-17

**Authors:** Syed Hussain Basha, R Nalini Prasad

**Affiliations:** 1 Dept. of Biotechnology, REVA Institute of Science and Management, Yelahanka, Bangalore 560 064, India

**Keywords:** Pleconaril, Oseltamivir, H1N1, Neuraminidase, Docking analysis

## Abstract

**Background:**

Neuraminidase (NA) is a prominent surface antigen of Influenza viruses, which helps in release of viruses from the host cells after replication. Anti influenza drugs such as Oseltamivir target a highly conserved active site of NA, which comprises of 8 functional residues (R118, D151, R152, R224, E276, R292, R371 and Y406) to restrict viral release from host cells, thus inhibiting its ability to cleave sialic acid residues on the cell membrane. Reports on the emergence of Oseltamivir resistant strains of H1N1 Influenza virus necessitated a search for alternative drug candidates. Pleconaril is a novel antiviral drug being developed by Schering-Plough to treat Picornaviridae infections, and is in its late clinical trials stage. Since, Pleconaril was designed to bind the highly conserved hydrophobic binding site on VP1 protein of Picorna viruses, the ability of Pleconaril and its novel substituted derivatives to bind highly conserved hydrophobic active site of H1N1 Neuraminidase, targeting which oseltamivir has been designed was investigated.

**Result:**

310 novel substituted variants of Pleconaril were designed using Chemsketch software and docked into the highly conserved active site of NA using arguslab software. 198 out of 310 Pleconaril variants analyzed for docking with NA active site were proven effective, based on their free binding energy.

**Conclusion:**

Pleconaril variants with F, Cl, Br, CH3, OH and aromatic ring substitutions were shown to be effective alternatives to Oseltamivir as anti influenza drugs.

## Background

**Figure 1:**
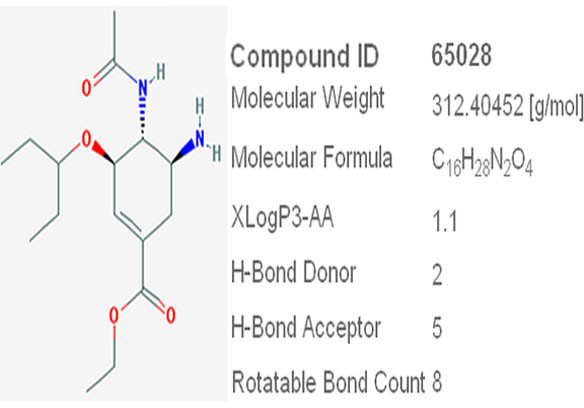
**Structure and properties of Oseltamivir**.

Most of the early antiviral drugs were discovered after screening large number of possible drug compounds using trial and error method. Lately, this approach has been largely replaced by rational drug design, in which, a target viral protein is identified for the drug [1]. A detailed picture of the 3 dimensional structure of the protein can be derived using *In-silico *computational techniques and a target site in the protein can be selected [2]. In influenza viruses, NA surface antigen plays a vital role in releasing the virus from the host cell during the budding stage [3]. Ever since the crystal structure of NA was determined, it is used as a target protein for many drug compounds. Oseltamivir (Figure 1) is one such approved anti-influenza drug compound that targets the highly conserved NA active site of H1N1 Influenza virus which comprises of 8 functional residues (R118, D151, R152, R224, E276, R292, R371 and Y406) [4,5]. The recent outbreaks of H1N1 and reports of oseltamivir resistant strains have necessitated the need to find effective alternatives to the existing anti-influenza drugs [6].

**Figure 2:**
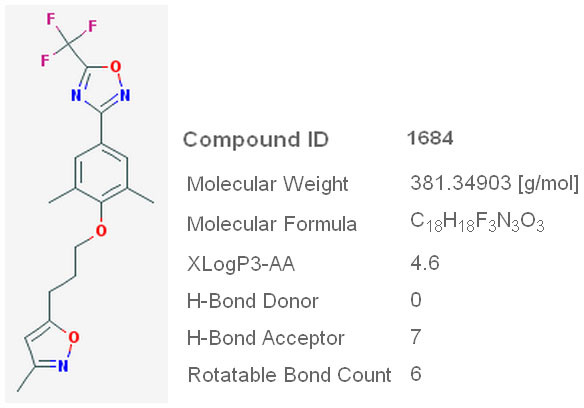
**Structure and properties of Pleconaril**.

Pleconaril (Figure 2) is a novel antiviral drug being developed by Schering-Plough to treat Picornaviridae infections, and is in its late clinical trials stage [7]. Highly conserved hydrophobic pocket of VP1 protein, which forms a part of picornaviral capsid, is the target for Pleconaril activity. In enteroviruses, this prevents the virus from exposing its RNA, and in rhinoviruses it prevents the virus from attaching itself to the host cell [8]. However, to date, to the best of our knowledge there is no study reporting the activity of Pleconaril against Influenza virus. Since, Pleconaril was designed to bind the highly conserved hydrophobic binding site on VP1 protein of Picorna viruses, the ability of Pleconaril and its novel substituted derivatives to bind highly conserved hydrophobic active site of H1N1 Neuraminidase, targeting which oseltamivir has been designed was investigated in this present study.

Molecular modeling tools help in generating new candidate drug molecules within a short span of time. After generation of new possible drug candidates, the drug and target protein interaction dynamics can be predicted by carrying out a docking analysis. The knowledge so derived is used to predict the strength of association or binding affinity between the two molecules based on scoring functions.

A database of potential drug molecules and target protein structure serve as inputs for docking analysis. The success of the docking analysis is determined by scoring function and search algorithm [9], which helps in determining the compatibility between the drug and its target protein. This technique is being used extensively to predict the geometries of different bimolecular complexes [10]. Scoring function predicts the strength of the binding affinity between ligand and the protein based on the complex geometry [11] and search algorithm analyzes the drug molecule for different binding positions with its target molecule, each binding position, which is termed a "pose", is used to generate the snapshot of interactions [12].

In our present study, a database of 310 novel substituted Pleconaril variants was built by altering the side chains and substituting different aromatic rings into the original Pleconaril molecule. Molecular docking analysis was performed to visualize the interaction of each of these variants with target molecule. An attempt was made to identify Pleconaril variants with best NA binding ability.

## Methods

### 1. Preparation of Receptor

The crystal structure of NA of 1918 Spanish flu (A/Brevig Mission/1/18 H1N1) virus (PDB ID: 3BEQ) was obtained from Protein Data Bank (PDB) [13] with a resolution factor of 1.64 Å and the method incorporated is X-Ray diffraction [14]. Before docking, the crystal structure of the protein was cleaned by removing the water molecules and hydrogen atoms were added to this target protein for correct tautomeric and ionization states of amino acid residues. The modified structure so obtained was saved in .pdb format and used for all docking studies.

### 2. Preparation of Ligands

**Figure 3:**
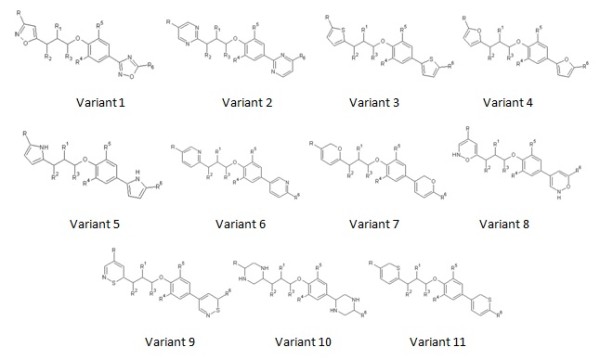
**11 Basic variants of Pleconaril**.

Oseltamivir (Compound ID 65028) [15] and Pleconaril (Compound ID 1684) [16] were obtained from pubchem database [17]. Using ACDLABS ChemSketch 11.0 [18] software, 310 novel substituted derivatives of Pleconaril were designed using Cl, F, Br, CH3 and OH functional groups as substitutes on R, R1, R2, R3, R4, R5, and R6 positions of 11 basic Pleconaril variants (Figure 3).

Using Arguslab 4.0.1 [19] software, hydrogen bonds were added to each molecule and fidelity of all bonds was checked using "add hydrogens" and "Clean Hybridization" options respectively. Geometry optimization was done using UFF [20-24] Molecular Mechanics (MM) method. Finally Oseltamivir, Pleconaril and all the novel substituted Pleconaril derivatives were saved in .mol format for further docking studies.

### 3. Determination of Active site

The highly conserved active site of NA, which comprises of 8 functional residues (R118, D151, R152, R224, E276, R292, R371 and Y406) targeting which oseltamivir has been designed was considered as active site for docking analysis.

### 4. Docking

Docking between receptor and ligands was performed using "Dock a Ligand" option of arguslab 4.0.1 software. A spacing of 0.4 Å between the grid points was used. "ArgusDock" was selected as docking engine. "Regular precision" was selected in docking precision menu, "Dock" was chosen as calculation type, "Flexible" for the ligand and "AScore" was used as the scoring function. A maximum of 150 poses were allowed to be analyzed, binding site box was set to 25 × 25 × 25 angstroms to encompass the entire active site. Each docking run was repeated three times to get best results. Resulted docked molecules were saved in .pdb format and all the docking images were generated using Accelrys^® ^Discovery Studio 3.0 Visualizer software [25].

## Results and discussion

All the 310 variants of Pleconaril molecule were analyzed for binding with the active site of NA. 198 out of these were found to have optimum binding efficiency, based on the binding energy calculations in comparison with Oseltamivir.

**Figure 4:**
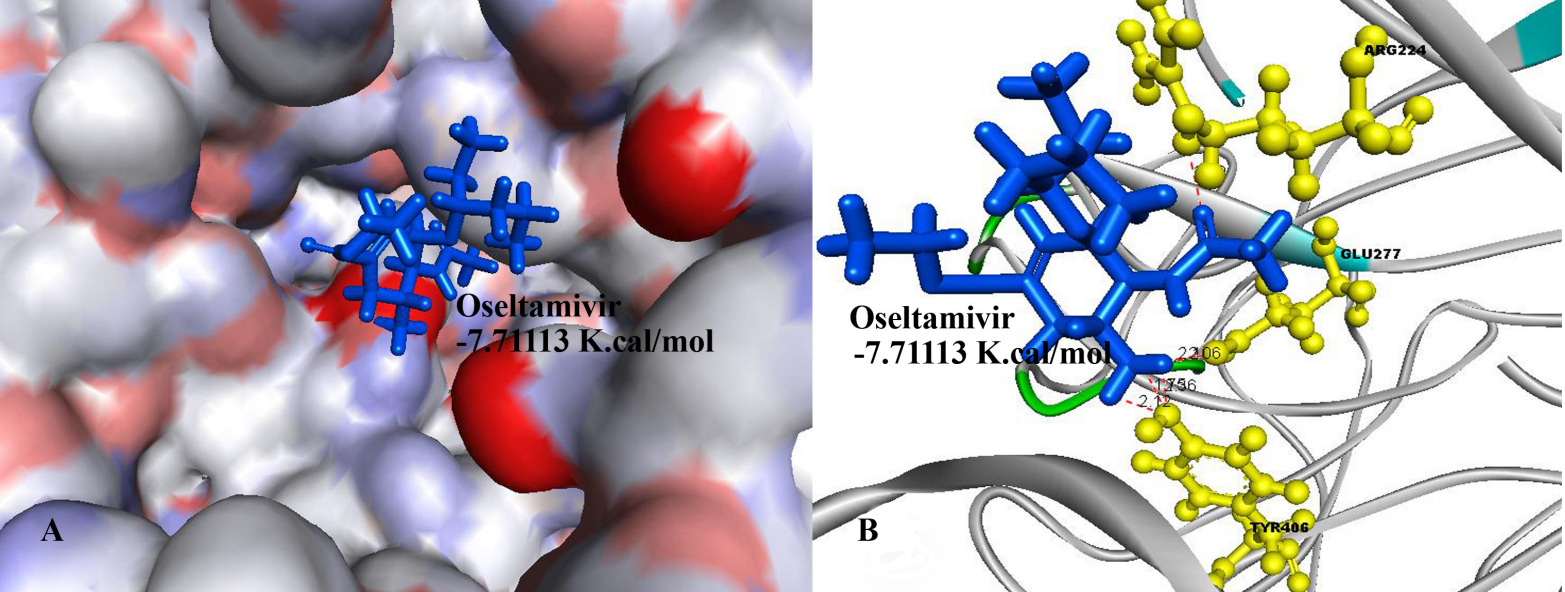
**A) Binding of oseltamivir (Blue) into the active site of NA**. B) Amino acid residues (Yellow) in the active site of NA interacting with Oseltamivir by hydrogen bonds (dotted red line) using -7.71113 K.Cal./mol. of binding energy.

**Figure 5:**
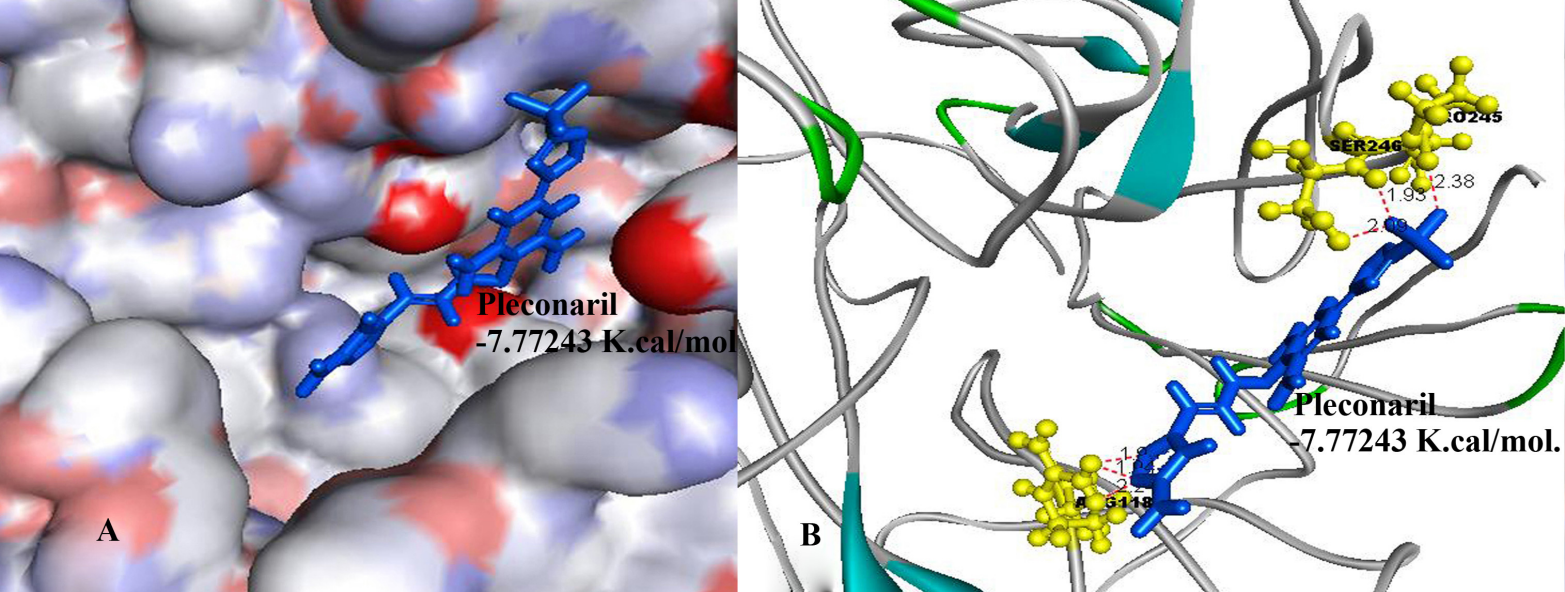
**A) Binding of Pleconaril (Blue) into the active site of NA**. B) Amino acid residues (Yellow) in the active site of NA interacting with Pleconaril by hydrogen bonds (dotted red line) using -7.77243 K.Cal./mol. of binding energy.

**Figure 6:**
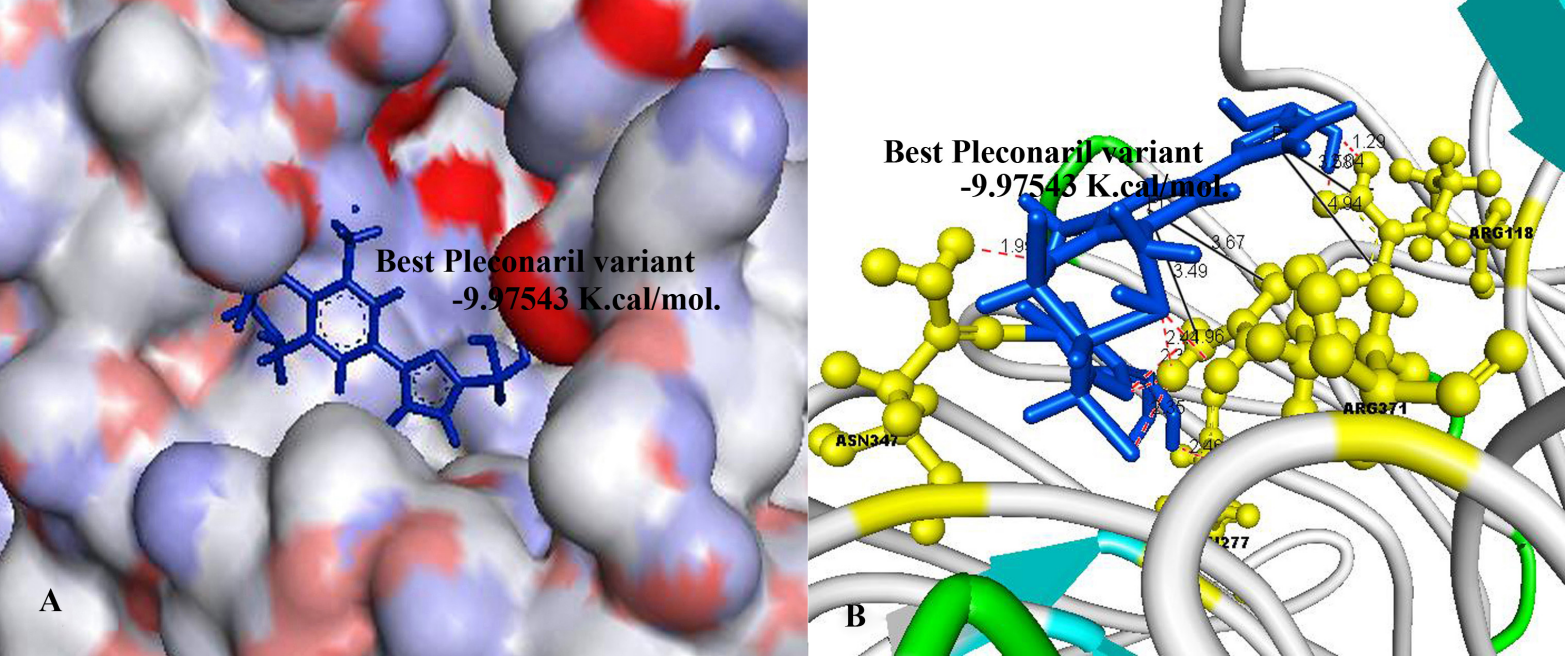
**A) Binding of Best Pleconaril variant (Blue) into the active site of NA**. B) Amino acid residues (Yellow) in the active site of NA interacting with Best Pleconaril variant by hydrogen bonds (dotted red line) and pi-cationic interactions (Solid Black line) using -9.97543 K.Cal./mol. of binding energy.

Further investigations showed that Oseltamivir formed 6 hydrogen bonds with TYR 406, GLU 277, ARG 224 (Figure 4) and Pleconaril formed 6 hydrogen bonds with SER 246, PRO 245, ARG 118 amino acid residues (Figure 5), whereas the best Pleconaril variant formed 9 hydrogen bonds with ARG 118, ASN 347, ARG 371 and GLU 277 amino acid residues of NA active site. Moreover the central benzene ring and furan ring of the best Pleconaril variant played a major role in stabilizing the ligand receptor complex by pi-cation interactions with amino acid residues ARG 118 and ARG 371 along with hydrogen bonds (Figure 6).

Pleconaril derivative (Numbered 3 in Table 1), with a binding energy of -9.97543 K.cal/mol. showed the least possible binding energy of all variants analyzed. Whereas the binding energy of oseltamivir, the original NA inhibitor, was found to be -7.7113 K.cal/mol. 44 variants of Pleconaril were found to be having binding energy around -9.0 K.cal/mol. 10 best variants out of these 44 were tabulated in Table 2. These molecules were selected because of their least binding energies, as the drug molecules with the least binding energy are generally considered to be having high binding efficiencies. Analysis of these 44 variants in further established that furan and thiophene substitutions along with the presence of central benzene ring in the main molecule played an important role in determining the binding affinity of the drug variants.

**Table 1:** Structure, molecular formula, binding energies of Oseltamivir, Pleconaril and best variant of pleconaril

S. No	Structure of molecule	Molecular formula	**Binding energy in K.cal./mol**.
1.		C_16_H_28_N_2_O_4 _	-7.71113

2.		C_18_H_18_F_3_N_3_O_3 _	-7.77243

3.		C_21_H_21_Cl_3_O_9 _	-9.97543

**Table 2:** Structure, molecular formula, binding energies of 10 best variants of pleconaril

S. No	Structure of molecule	Molecular formula	**Binding energy in K.cal./mol**.
1.		C_21_H_21_Cl_3_O_4_S_2_	-9.90775

2.		C_21_H_21_F_3_O_7_S_2_	-9.90392

3.		C_21_H_21_Br_3_O_7_S_2_	-9.90239

4.		C_21_H_21_BrClFO_7_S_2_	-9.69162

5.		C_23_H_25_BrClFO_7_S_2_	-9.67661

6.		C_21_H_21_Cl_3_O_7_S_2_	-9.60862

7.		C_21_H_21_Br_3_O_9_	-9.57821

8.		C_23_H_28_OS_2_	-9.52213

9.		C_23_H_19_Br_6_Cl_3_OS_2_	-9.47199

10.		C_23_H_19_BrCl_7_FOS_2_	-9.46511

## Conclusion

Computer aided drug designing and molecular docking analysis are highly effective in creating and analyzing new candidate drug molecules. 198 out of 310 Pleconaril variants analyzed for docking with NA active site were proven effective. Pleconaril variants with F, Br, CH3, Cl, OH and aromatic ring substitutes showed higher levels of NA binding ability. Several interactions such as hydrogen bonds, hydrophobic, hydrophilic interactions, electrostatics and Vanderwaal forces are thought to have played an important role in stabilizing the drug and target complexes based on the theoretical modeling. Thus, based on the above results we propose Pleconaril variants numbered 3, 4 and 11 (Figure 4) with F, Br, CH3, Cl and OH substitutions at R, R1, R2, R3, R4, R5, and R6 positions have a definite potential to be developed as lead compounds for H1N1 Influenza virus. However as it is only a preliminary *In-silico *investigation in modifying the Pleconaril molecule for anti-influenza activity, further *In-vivo *validation and conformation of the present findings is required.

## Competing interests

The authors declare that they have no competing interests.

## Authors' contributions

SHB conceptualized the idea, carried out the work including acquisition of the data, analysis of the final results and drafting the manuscript. RNP was involved in supervising the work designed, analysis and interpretation of the data as well as drafting the manuscript. Both authors read and approved the final manuscript.
